# Multi-omics integration in allergy, viral infections and asthma: a comprehensive analysis of bioinformatics tools

**DOI:** 10.3389/fbinf.2026.1904588

**Published:** 2026-09-22

**Authors:** Silvia Garcés Rigol, Nuria Contreras, Rafael Núñez, Elena Izquierdo, Domingo Barber, Alma Villaseñor, Osvaldo Graña Castro, María M. Escribese

**Affiliations:** 1 Departamento de Ciencias Médicas Básicas, Instituto de Medicina Molecular Aplicada – Nemesio Díez (IMMA-ND), Facultad de Medicina, Universidad San Pablo-CEU, CEU Universities, Boadilla del Monte, Spain; 2 Centro de Metabolómica y Bioanálisis (CEMBIO), Facultad de Farmacia, Universidad San Pablo-CEU, CEU Universities, Boadilla del Monte, Spain

**Keywords:** allergy, asthma, inflammatory respiratory diseases, integration, multi-omics, performance

## Abstract

**Introduction:**

Allergic and inflammatory respiratory conditions are complex and heterogeneous diseases affecting a large portion of the population. Although omics technologies have enabled the identification of potential biomarkers, single-omics-based approaches often fail to capture the full complexity of these disorders. Multi-omics integration provides a more comprehensive perspective; however, selecting the most suitable tool remains challenging due to the wide range of available tools and the variability of biological datasets. In this study, we compare different integration approaches for these diseases, evaluating their performance and functionality to ensure a robust and objective assessment across heterogeneous datasets.

**Methods:**

We compared multiple multi-omics integration tools across four heterogeneous respiratory disease datasets. Unsupervised methods were applied following dataset-specific preprocessing, including filtering, imputation, and normalization. Integration was performed across matched metabolomics, proteomics, and transcriptomics layers. Performance was evaluated using the Adjusted Rand Index (ARI).

**Results:**

Unsupervised methods showed variable performance across datasets. NEMO consistently achieved the most accurate clustering of clinical phenotypes, whereas MOFA and intNMF performed well in specific contexts.

**Conclusion:**

Our findings indicate that NEMO, intNMF and MOFA are promising tools for multi-omics integration in allergic and respiratory diseases, as they effectively capture complementary information across different omics layers and facilitate the identification of coordinated molecular signatures associated with disease phenotypes. Overall, these results emphasize the importance of selecting integration methods based on the study goals and the dataset characteristics.

## 1 Introduction

Allergic diseases arise from dysregulated immune responses to environmental antigens (Dougherty et al., 2026) and are influenced by complex genetic and environmental interactions (Vogeser and Bendt, 2023). Thus, for a broader understanding of the mechanisms underlying allergies, high-throughput strategies provide a clear advantage, enabling system-level analysis. Omics techniques make it possible to obtain large amounts of data at different molecular levels from a biological sample, providing a holistic view of biological systems (Vogeser and Bendt, 2023). Due to the increased number of studies focused on omics analyses, precision medicine is seeing an improvement in disease diagnosis, treatment tailoring, and prognosis prediction (Tebani et al., 2016). The need to identify new allergic disease-related biomarkers continues to grow. Omics analyses provide valuable clinical insights and contribute to several applications, including patient stratification (Bodaghi et al., 2023) and treatment monitoring (Contreras et al., 2024). Although the analysis of individual omics datasets can elucidate certain disease mechanisms in complex diseases, such as inflammatory diseases and allergies, integrating multiple omics layers is essential to avoid overlooking crucial interactions across different molecular levels (Tini et al., 2019). Integrating information from individual omics datasets can provide a more comprehensive understanding of the complexity underlying biological systems (Santiago-Rodriguez and Hollister, 2021). Therefore, systems biology aims to integrate these datasets to identify molecular networks associated with different patient states and disease phenotypes (Pinu et al., 2019).

There are five major omics technologies: genomics, epigenomics, transcriptomics, proteomics, and metabolomics (Vogeser and Bendt, 2023). Several studies have shown the utility of these omics’ platforms in allergy research. For example, variants in the SERPINB gene have been linked with food allergies (Marenholz et al., 2017), while ORMLD3 polymorphisms are associated with severe asthma in childhood (Schedel et al., 2015). Integrated transcriptomic and metabolomic analyses in patients with urticaria revealed differential expression of IL-22-related mRNAs and significantly reduced salicylic acid levels in desensitized patients (Tay et al., 2022). In line with these findings, our group has also identified a dysregulation of sphingolipid metabolism linked to severe allergic phenotypes (Contreras et al., 2024; Delgado-Dolset et al., 2021; Obeso et al., 2018; Couto-Rodriguez et al., 2023; Pablo‐Torres et al., 2023). Thus, the integration of different omics layers, such as transcriptomics, proteomics, and metabolomics, has led to the identification of potential biomarkers and has contributed to the elucidation of novel mechanisms underlying these diseases (Saito et al., 2024; Devonshire et al., 2023).

Despite the growing interest in multi-omics approaches, multi-omics data integration remains a relatively recent field. Furthermore, the absence of standardized integration protocols continues to pose significant challenges. This has led to substantial variability in analytical approaches, which directly influences biological interpretation and the identification of clinically relevant biomarkers (Mohr et al., 2024). The lack of standardization mainly arises from the heterogeneity of data sources (i.e., differences in the number of variables and measurement types) and the diverse techniques used to generate these datasets, resulting in variations in scale, structure, and data distribution (Picard et al., 2021). Additionally, the contribution of each omics layer to the analysis may vary, as some layers provide clearer biological insights, whereas others are more difficult to interpret due to noise, sparsity, or limited annotation (Jung et al., 2020).

Accordingly, selecting the most appropriate integration method remains a major challenge, as no single approach performs optimally across all scenarios. The choice depends on multiple factors, including the research objective (i.e., unsupervised and supervised analysis), data heterogeneity (e.g., presence of missing values, differences in sample size, and variations in scale), and the available computational resources (Tarazona et al., 2021). In line with the study hypothesis, unsupervised models are primarily used for exploratory data analysis, including the clustering of subjects and the determination of the most relevant features contributing to biological variability that could explain predefined disease classifications. These approaches can also facilitate the identification of novel phenotypes or endotypes. In contrast, supervised methods aim to develop predictive models based on pre-defined clinical labels, either as classification tools or as feature-selection strategies for the discovery of new biomarkers.

Despite its potential, multi-omics integration remains underexplored in allergic diseases compared to fields such as oncology, where it has been more widely applied, generating robust evidence for biomarker discovery and providing valuable mechanistic insights (Llinas-Bertran et al., 2025; Cai et al., 2022; Sathyanarayanan et al., 2020; Nicora et al., 2020). In the field of allergic diseases, determining how different multi-omics integration tools perform across diverse datasets remains an unanswered question. Therefore, a comprehensive evaluation of the main available unsupervised integration strategies is needed, considering variations in data type, sample size, and the number of omics layers involved. Thus, in this study, we compare unsupervised bioinformatic tools for the integration of multi-omics datasets related to respiratory diseases, evaluating their methodological features and performance using different metrics and parameters to ensure robust and objective assessment.

## 2 Materials and methods

### 2.1 Datasets used for comparisons

To address the potential diversity of data in the immune-related research field, four different representative datasets on inflammatory respiratory diseases were selected, each with different features in terms of the (i) disease; (ii) nature and size of the dataset (number of patients and groups involved); and (iii) an established phenotypic classification. All datasets used in this study are publicly accessible:

1. COVID-19 dataset (COVID-19) (Su et al., 2020): plasma samples from healthy donors (not included in this study) and 263 patients with ongoing COVID-19 infection were taken at two different timepoints (after initial diagnosis (T1) and days after (T2)). Samples were categorized using the World Health Organization (WHO) ordinal score (WOS) of disease severity (World Health Organization, 2020). Therefore, the COVID-19 samples were classified as mild (n = 109), moderate (n = 102) and severe (n = 52). The omics included in this dataset are the following (Data is accessible at https://atlas.fredhutch.org/isb/covid/):
  - Targeted proteomics: 92 proteins from Olink® Bioscience were analyzed by five different panels, obtaining data for 454 proteins.
  - Untargeted metabolomics: ultra-high-performance liquid chromatography coupled with tandem mass spectrometry (UHPLC-MS/MS) was used, resulting in the identification of 1,050 metabolites.
2. Long-term effects of COVID-19 dataset (Long COVID-19) (Wang et al., 2023): plasma samples were taken from 28 healthy subjects (controls) and 117 patients who had a COVID-19 infection. Long COVID-19 samples were collected at two timepoints: in the acute phase and 6 months later, in the convalescence phase, to assess differences in molecular characteristics. Cytokines, proteins and metabolites were analyzed:
  - Targeted cytokines: 47 cytokines were measured using Luminex xMAP technology.
  - Targeted proteomics: liquid chromatography coupled to mass spectrometry in multiple reaction monitoring mode (LC-MRM-MS) was used to obtain proteomic profiles for the samples. A total of 274 proteins were quantified. Raw data can be accessed in PeptideAtlas (ID: PASS03810).
  - Targeted metabolomics: a custom-made approach of a combination of direct infusion (DI-MS) and LC-MS/MS was used to identify and quantify a total of 635 metabolites (MetaboLights ID: MTBLS7337).
3. Severe Asthma dataset (Contreras et al., 2024): Serum samples from severe asthmatic patients classified following the GINA guidelines to be treated with Mepolizumab (pre-Mepolizumab; n = 18) or Omalizumab (pre-Omalizumab; n = 16) were collected before treatment initiation. Two different omics datasets were generated:
  - Targeted proteomics: 45 proteins were quantified with the Olink® Target 48 Cytokine panel.
  - Targeted metabolomics: 36 metabolites were quantified using a LC coupled to triple quadrupole MS (LC-QqQ-MS). Accessible at DOI: 10.21228/M8SB0G.
4. Grass-pollen respiratory allergy dataset (Grass-pollen allergy) (Obeso et al., 2018): A cohort of patients with grass pollen allergy was recruited and classified following clinical evaluation into three severity groups: mild (n = 5), moderate (n = 5), and severe (n = 8). In addition, non-allergic individuals were included as healthy controls (n = 6). Two omics were used to study the molecular profile of these patients:
  - Untargeted metabolomics: 107 metabolites were reported by using a LC-MS and gas chromatography coupled to MS (GC-MS). Accessible at DOI: 10.21228/M82Q3W.
  - Transcriptomics: Affymetrix expression microarrays obtained data for 48,226 genes (GSE114707).

A summary of the characteristics of each dataset can be found in Figure 1.

**FIGURE 1:**
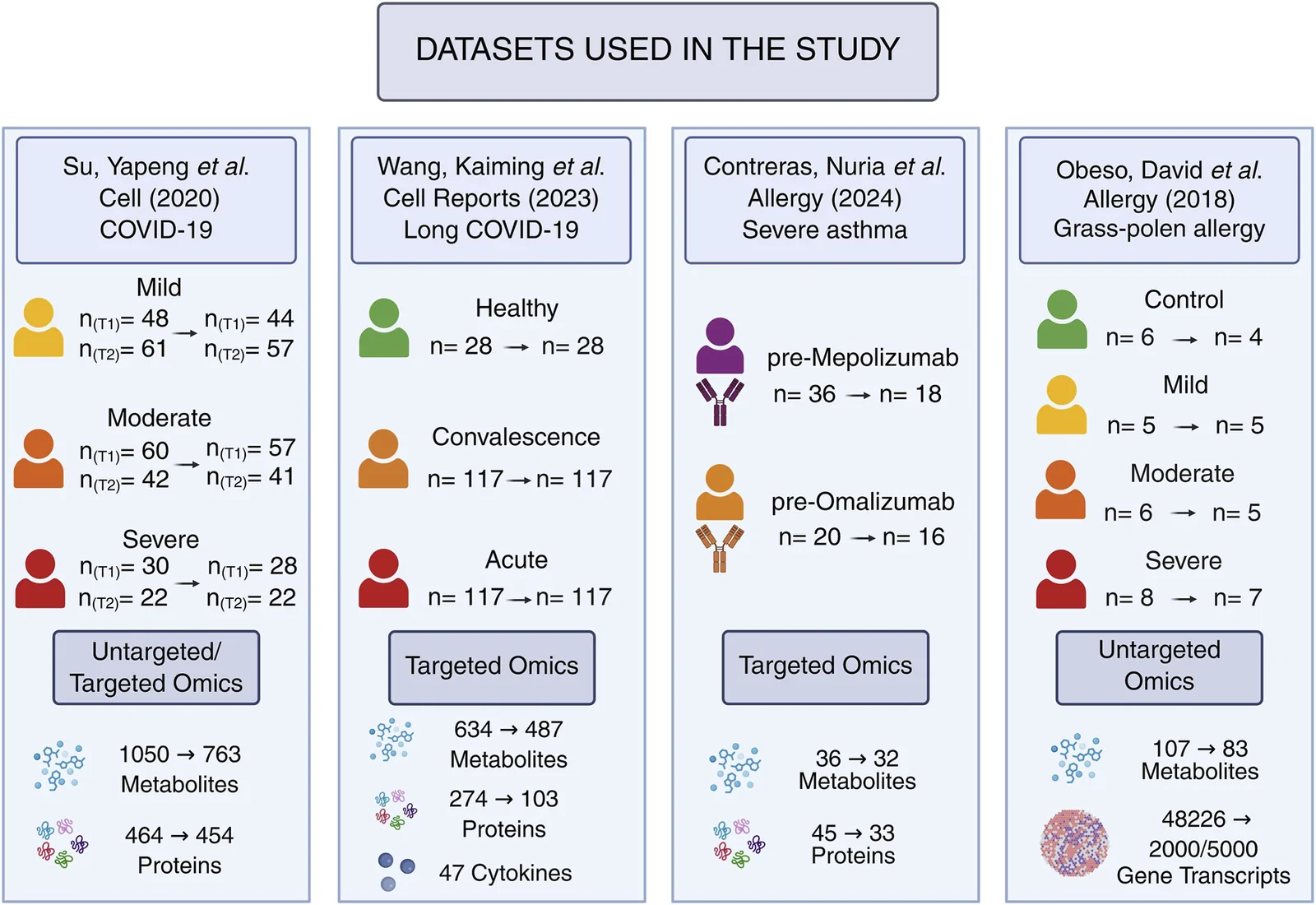
Summary of the datasets used to test the different integration tools. Each dataset contains information on how the samples are classified and which omics have been analyzed. Sample and variable sizes after pre-processing.

### 2.2 Data preprocessing

Data was preprocessed prior to analysis using dataset-specific approaches to account for differences in data characteristics and complexity (Figure 1). Missing data thresholds were selected considering the characteristics of each omics layer. Since transcriptomic, proteomic, and metabolomic datasets differ substantially in feature dimensionality, abundance distributions, and missing-value generation mechanisms, a single threshold was not considered appropriate for all data types. The chosen criteria aimed to balance feature retention with data reliability, limiting the influence of heavily incomplete variables while preserving sufficient biological information for integrative analysis. Additional filtering scenarios were evaluated as part of the robustness assessment and are presented in Supplementary Figure 1.

- COVID-19: Metabolites with more than 20% missing values were removed (Hendrickx et al., 2025); the remaining ones were imputed using the K-Nearest Neighbors (KNN) algorithm, resulting in 763 metabolites (Kashyap, 2024). For the proteomics dataset, proteins with more than 15% missing values across samples were removed to maintain a comparable number of features between datasets, resulting in 454 proteins. Subjects with at least one missing value in the proteomic dataset were excluded from the analysis. Two time points were subsequently analyzed.
- Long COVID-19: For the metabolomics dataset, metabolites with missing values or values below the limit of detection were removed, resulting in 487 metabolites. For the proteomics dataset, proteins with missing values or with >35% of measurements below the limit of detection were removed to ensure a sufficient number of analyzable features. For the remaining proteins, values below the limit of quantification were imputed to the minimum value, resulting in 103 proteins. For the cytokine dataset, the only missing value was imputed using the KNN algorithm (Kashyap, 2024).
- Severe Asthma: No missing values were found for the metabolomics dataset, whereas for the proteomics dataset, missing values had already been imputed by the original authors using the KNN algorithm (Contreras et al., 2024). Only pre-treatment samples were retained for analysis.
- Grass-pollen allergy: No missing values were detected in either dataset. For the metabolomics data, duplicate metabolites were removed, resulting in 83 retained metabolites. Due to the high dimensionality of the transcriptomics data, gene covariance was calculated and used to rank genes. Following a previously established methodology (Hasanzadeh and Charkari, 2025), the top 2,000 and top 5,000 genes with the highest covariance values were selected for downstream analysis.

After these pre-processing steps, the omics datasets were subsetted to retain only samples from the same individuals within each cohort study. Subsequently, only the metabolomics and Luminex-based proteomics datasets were normalized using Z-score normalization (Henderi, 2021), as the transcriptomics and Olink®-based proteomics data had already been normalized.

### 2.3 Data integration tools

To ensure a comprehensive and unbiased evaluation, the integration tools included in this study were selected to represent different methodological approaches. These approaches comprise dimensionality reduction (DR) techniques, including matrix factorization (MF); Bayesian methods based on probabilistic modelling (Vahabi and Michailidis, 2022), and similarity-based approaches, which compute distances among the data (Zhang et al., 2022) (Table 1).

**TABLE 1:** Summary of the characteristics of the integration tools used in this study.

Method	Methodological approach	Machine learning type
SNF	Similarity	Unsupervised
NEMO	Similarity	Unsupervised
MCIA	DR	Unsupervised
moCluster	DR/MF	Unsupervised
PINSPlus	Similarity	Unsupervised
intNMF	DR/MF	Unsupervised
MOFA	MF/Bayesian	Unsupervised
sPLS	DR	Unsupervised

intNMF, Integrative non-negative matrix factorization; MCIA, Multiple Co-inertia Analysis; MF, matrix factorization; MOFA, Multi-Omics Factor analysis; NEMO, Neighbourhood-based multi-omics clustering; PINSPlus, Perturbation clustering for data INtegration and disease Subtyping; SNF, similarity network fusion; sPLS, sparse Partial Least Squares.

The selected methods are:

- Similarity Network Fusion (SNF): integrates multi-omics data using a network fusion approach based on similarity matrices (Subramanian et al., 2020). To do so, a patient similarity matrix is first constructed for each omics layer and subsequently fused into a single network using a KNN algorithm (Kashyap, 2024).
- Neighborhood-based multi-omics clustering (NEMO): integrates multi-omics using a similarity-based approach (Subramanian et al., 2020). Patient similarity matrices are generated from Euclidean distances within each omics layer and then combined to identify common clusters across datasets (Rappoport et al., 2019).
- Multiple Co-inertia Analysis (MCIA): performs an exploratory data analysis to identify relationships and concordance among datasets by creating a set of vectors that contain the sum of the squared covariances (Min and Long, 2020; Pierre-Jean et al., 2020).
- moCluster: identifies latent variables using sparse consensus PCA (CPCA) (Meng et al., 2016), enabling the modelling from different groups and posterior mapping to different omics layers (Pierre-Jean et al., 2020). The resulting variables are then clustered using hierarchical clustering or K-means (Meng et al., 2016).
- Perturbation clustering for data INtegration and disease Subtyping (PINSPlus): integrates multi-omics data by constructing patient similarity graphs for each omics layer and derives a fused similarity matrix based on graph connectivity. This integrated matrix is subsequently used to identify robust patient subtypes through clustering analysis (Nguyen et al., 2019).
- Integrative non-negative matrix factorization (intNMF): integrates multi-omics data by imposing non-negative constraints and decomposing the input data matrices into shared non-negative factors, which are subsequently used for sample clutering (Pierre-Jean et al., 2022).
- Multi-Omics Factor Analysis (MOFA): extracts latent factors from multi-omics datasets to reduce data dimensionality and capture the main sources of variability (Argelaguet et al., 2020). It relies on a probabilistic Bayesian approach to infer latent factors and their associated weighting matrices (Argelaguet et al., 2018).
- mixOmics: an R package that provides several methods for multi-omics data integration (Subramanian et al., 2020). For unsupervised analyses (s)PLS (sparse Partial Least Squares) is used to identify linear combinations of variables across datasets, thereby reducing data dimensionality and capturing the major sources of variation.

### 2.4 Metrics used for evaluation

To enable an objective comparison across integration tools and datasets, appropriate evaluation metrics were used. The Rand Index and the Adjusted Rand Index (ARI) were calculated (GeeksforGeeks, 2025). This metric quantifies the similarity between two groupings (predefined clinical phenotype labels and the clusters obtained through unsupervised clustering). They calculate the proportion of pairs of subjects that are equally placed together or separated in both groupings. As the Rand Index does not consider correct pairing by chance, ARI was developed to address this issue by accounting for random clustering that results in non-zero Rand-Index values. ARI values range from −1 to 1, where 1 indicates perfect agreement, 0 corresponds to random clustering, and negative values indicate agreement lower than expected by change. Additionally, a permutation test was performed to assess the statistical significance of the observed ARI values (Qannari et al., 2014).

## 3 Results

Here, we present the results obtained from the application of unsupervised algorithms in each cohort.

### 3.1 COVID-19 severity is best clustered by MOFA

We first analyzed the COVID-19 dataset, which included patients with mild, moderate, and severe symptoms at two timepoints. At baseline (T1), unsupervised clustering analysis revealed that NEMO, SNF, and MOFA achieved the highest ARI values (>0.450), showing superior performance in grouping samples according to clinical severity (Figure 2A). The heatmaps generated by NEMO and SNF clearly separated mild cases into cluster 1, while moderate and severe cases were grouped into cluster 2 (Figure 2B; Supplementary Figure 2A). Using the latent factors explaining the highest proportion of variance, MOFA identified three clusters, with cluster 1 and cluster 3 mostly corresponding to mild and severe clinical categories, respectively, whereas cluster 2 consisted mainly of moderate and severe cases (Figure 2D).

**FIGURE 2:**
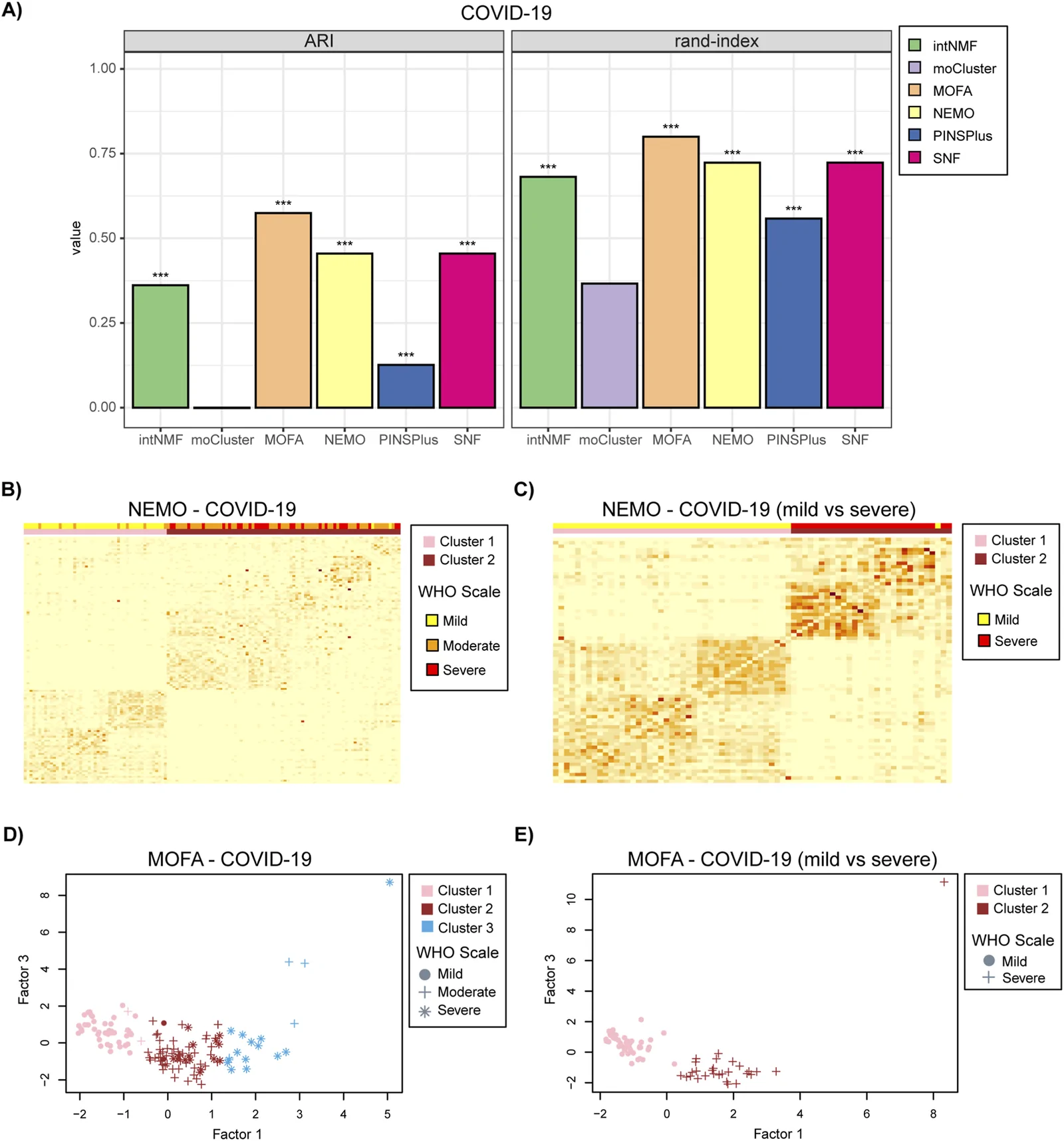
Results of the COVID-19 dataset at T1. **(A)** ARI values, p-values (* <0.05; ** <0.01; *** <0.001), and RI values obtained for each integration tool. NEMO similarity heatmaps for **(B)** Mild, Moderate and Severe, and **(C)** Mild and Severe clinical groups. Each row and column represents a sample, and color intensity corresponds to pairwise sample similarity derived from Euclidean distances, with darker colors (red) indicating greater similarity. MOFA plots of samples in latent factors for **(D)** Mild, Moderate and Severe, and **(E)** Mild and Severe clinical groups.

When the analysis was restricted to the extreme groups (mild vs. severe), NEMO, SNF, and MOFA demonstrated a clear separation between the two groups (Figures 2C,E; Supplementary Figure 2B). Additional results for the different methods are shown in Supplementary Figures 2, 3 for T1 and T2, respectively.

Overall, MOFA showed the best performance across timepoints (T1: ARI = 0.574; p-value <0.001; and T2: ARI = 0.540; p-value <0.001), achieving superior sample clustering and capturing biologically relevant differences associated with disease severity. These results suggest that disease severity is more clearly captured in extreme clinical phenotypes.

### 3.2 In Long COVID-19, NEMO identifies clinically meaningful clusters

Next, we examined the Long COVID-19 dataset, comparing control, acute, and convalescence groups. Here, NEMO and PINSPlus achieved the highest clustering performance (ARI values > 0.492; Figure 3A). NEMO accurately separated the three clinical groups, assigning control samples to cluster 2, most acute samples to cluster 1, and most convalescence samples to cluster 3 (Figure 3B). In contrast, PINSPlus grouped acute samples predominantly in cluster 2, whereas control and convalescence samples were distributed within cluster 1 (Figure 3C). intNMF showed weaker separation with convalescence samples distributed across all three clusters (Figure 3D). Similarly, MOFA exhibited comparable performance (Figure 3E). SNF, moCluster, MCIA and sPLS results are shown in Supplementary Figure 4.

**FIGURE 3:**
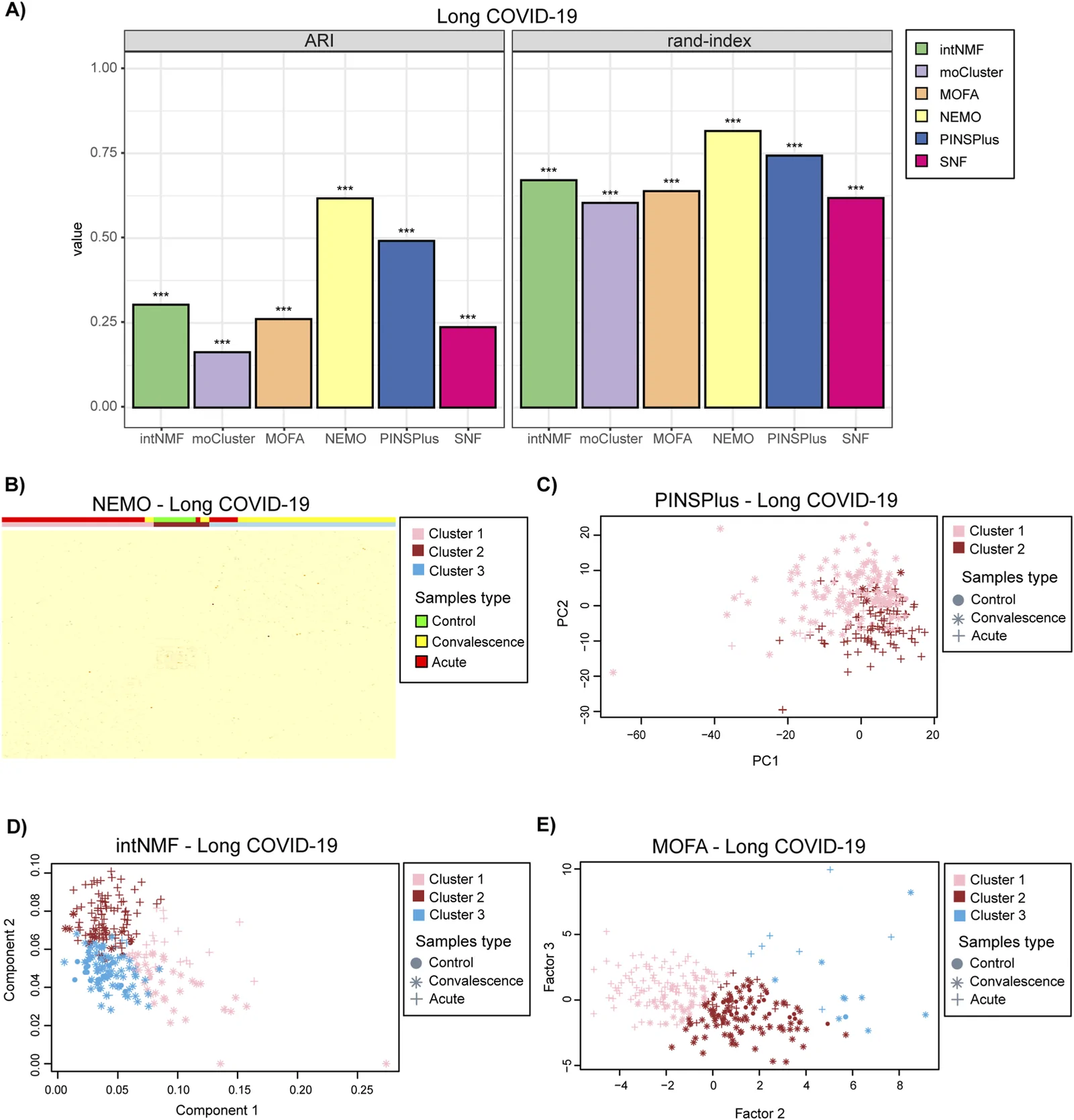
Results of the Long COVID-19 dataset for the Control, Convalescence and Acute clinical groups. **(A)** ARI values, p-values (* <0.05; ** <0.01; *** <0.001), and RI values obtained for each tool. **(B)** NEMO heatmap. Each row and column represents a sample, and color intensity corresponds to pairwise sample similarity derived from Euclidean distances, with darker colors (red) indicating greater similarity. **(C)** PINSPlus plot **(D)** intNMF plot, and **(E)** MOFA plot.

Overall, NEMO outperformed the other integration methods (ARI = 0.617, p-value <0.001), generating clusters that closely aligned with the clinical groups and more effectively captured the underlying heterogeneity of Long COVID-19.

### 3.3 IntNMF reveals hidden subphenotypes in severe asthma

We then focused on the Severe Asthma dataset, comparing patients clinically selected to receive Mepolizumab (pre-Mepolizumab) or Omalizumab (pre-Omalizumab). Among the evaluated methods, intNMF achieved the highest performance (ARI >0.5; p-value <0.050; Figure 4A; Supplementary Figure 1). However, it separated the two clinical groups into four clusters: pre-Mepolizumab patients were mainly distributed across clusters 1 and 2, whereas pre-Omalizumab patients were predominantly assigned to cluster 3. Cluster 4 contained a mixture of patients from both groups (Figure 4B), suggesting the presence of additional biological heterogeneity beyond the established clinical classifications.

**FIGURE 4:**
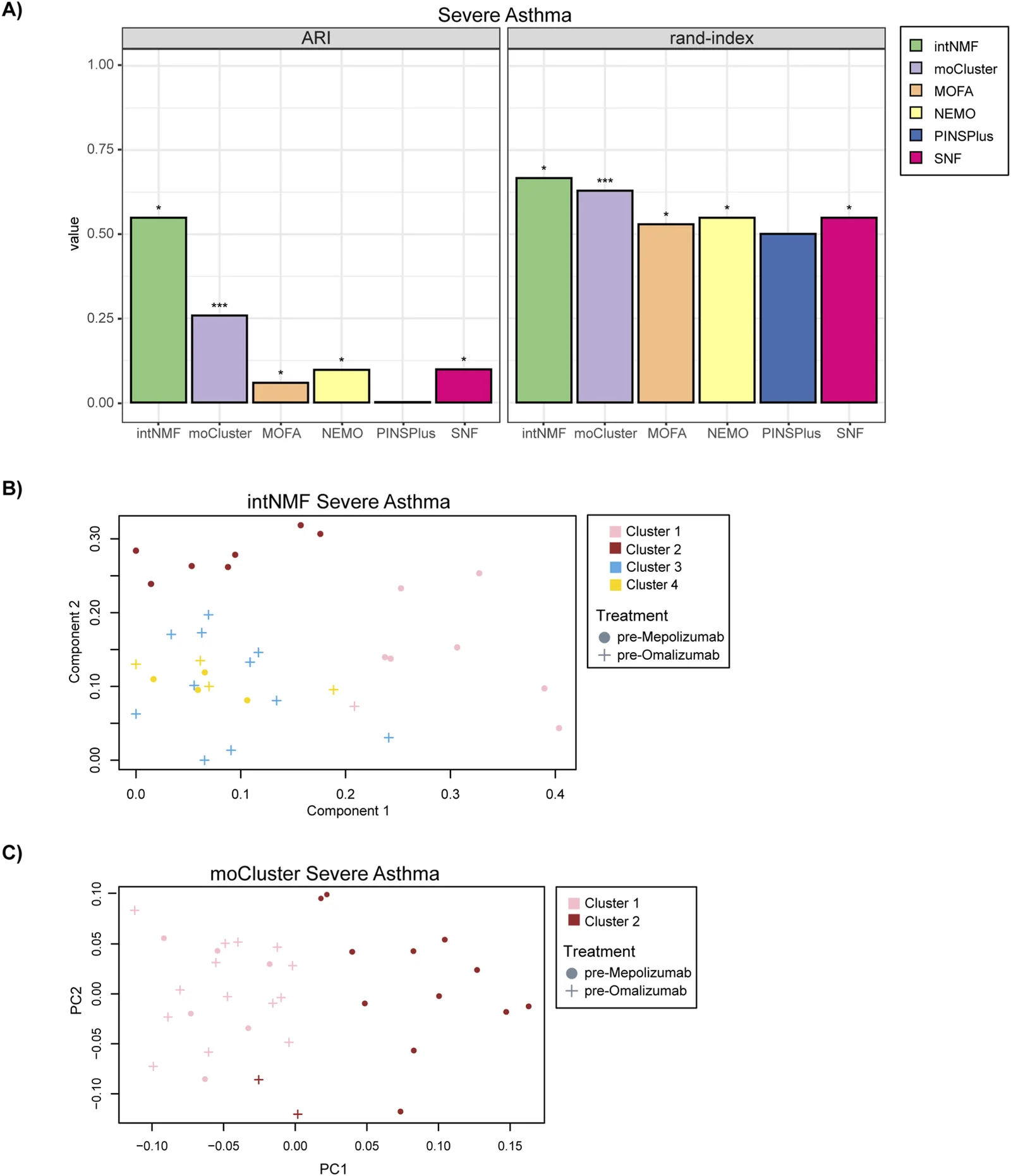
Results of the Severe Asthma dataset for pre-Mepolizumab and pre-Omalizumab groups. **(A)** ARI values, p-values (* <0.05; ** <0.01; *** <0.001), and RI values obtained for each integration tool. **(B)** intNMF plot **(C)** moCluster plot.

The second-highest ARI and the lowest p-value were obtained with moCluster (ARI = 0.268; p-value <0.001), which separated pre-Mepolizumab and pre-Omalizumab patients into two clusters, although some misclassifications were noted, particularly within the pre-Mepolizumab group (Figure 4C). The remaining methods showed lower clustering performance (ARI <0.259; Supplementary Figure 5).

In this instance, moCluster showed the closest agreement with the clinical group allocation and yielded the lowest p-value among the evaluated methods, although intNMF achieved the highest ARI and identified a more defined clustering structure.

### 3.4 moCluster and PINSPlus capture clinically relevant stratification in grass-pollen allergy

Finally, we analyzed **t**he Grass-pollen allergy dataset, comprising control, mild, moderate, and severe samples. Using the top 5,000 genes, most integration methods showed similar performance (ARI >0.30; p-value <0.001; Figure 5A), with NEMO, moCluster, SNF and PINSPlus, while MOFA showed lower consistency (ARI = 0.164; p-value <0.05).

**FIGURE 5:**
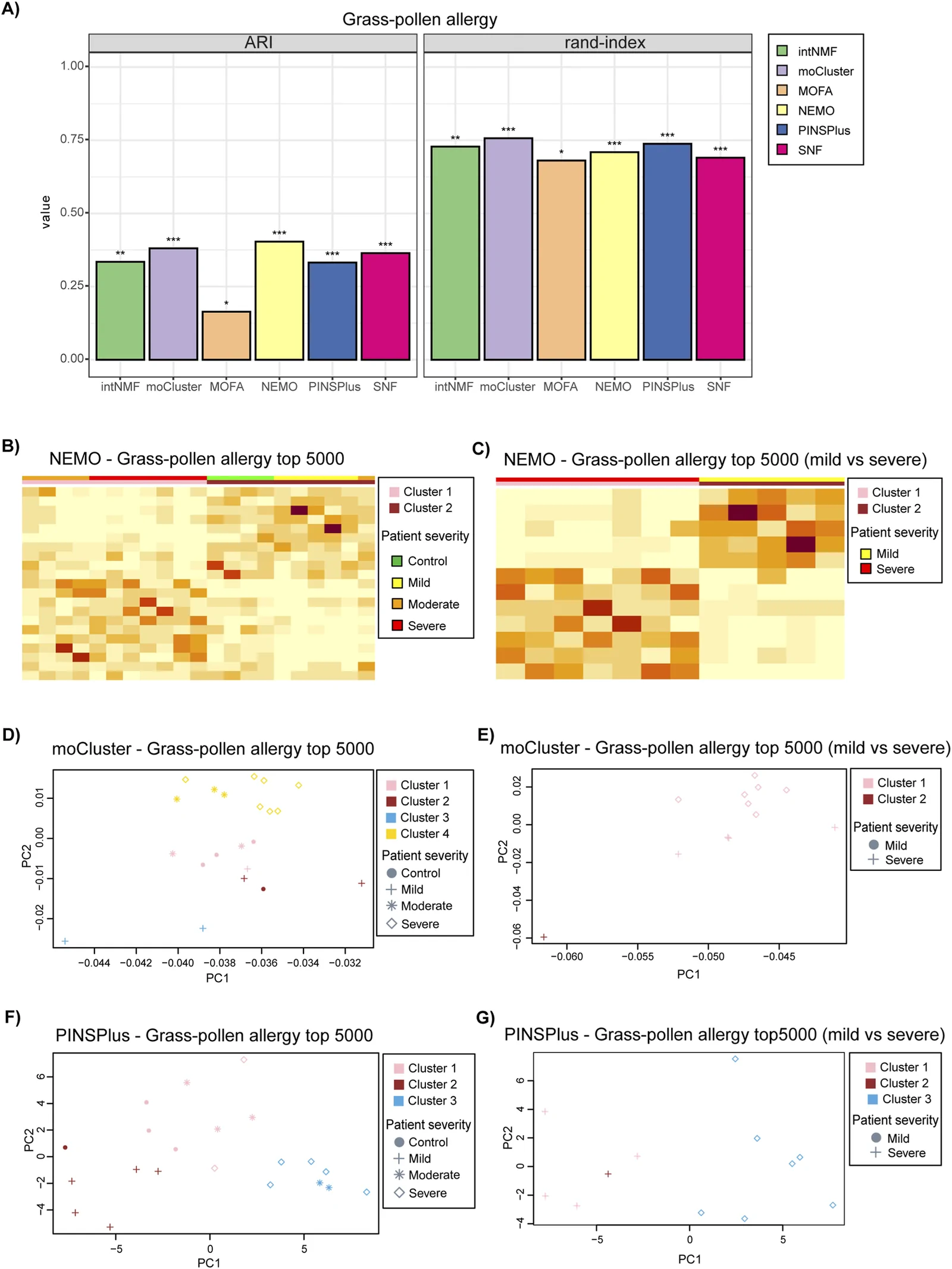
Results of the Grass-pollen allergy top 5,000 dataset. **(A)** ARI values, p-values (* <0.05; ** <0.01; *** <0.001), and RI values obtained for each method. NEMO heatmaps for **(B)** Control, Mild, Moderate and Severe, and **(C)** Mild and Severe clinical groups. Each row and column represents a sample, and color intensity corresponds to pairwise sample similarity derived from Euclidean distances, with darker colors (red) indicating greater similarity. moCluster plots for **(D)** Control, Mild, Moderate and Severe, and **(E)** Mild and Severe clinical groups. PINSPlus plots for **(F)** Control, Mild, Moderate and Severe, and **(G)** Mild and Severe clinical groups.

NEMO and SNF divided samples into two clusters: one containing control and mild cases and another containing moderate and severe cases (Figure 5B; Supplementary Figure 6A), differing only in the assignment of a single moderate patient. When the analysis was restricted to extreme phenotypes, both methods achieved perfect separation, highlighting clear differences in omics profiles between mild and severe cases (Figure 5C; Supplementary Figure 6B).

moCluster identified a cluster enriched for the most severe phenotypes (moderate and severe cases), although the remaining clusters showed substantial mixing across clinical groups (Figure 5D). Its performance was more limited in the mild-versus-severe comparison (Figure 5E). In contrast, PINSPlus identified three clusters with partial correspondence to clinical severity, assigning mild samples predominantly to cluster 2, most severe samples to cluster 3, and the remaining samples to cluster 1 (Figure 5F). In the extreme group comparison, PINSPlus effectively separated the samples, with only one mild sample misassigned to cluster 2 (Figure 5G). The remaining methods obtained similar results (Supplementary Figures 6C,D).

Additionally, the results using the top 2000 genes closely resemble those for the top 5,000 genes (Supplementary Table 7).

For the Grass-pollen allergy dataset using the top 5,000 genes, the evaluated methods were unable to clearly separate patients into the four clinical groups. However, NEMO (ARI = 0.403; p-value <0.001) and SNF (ARI = 0.364; p-value <0.001) captured biologically meaningful stratification associated with disease severity, despite failing to recover a four-class structure fully consistent with the clinical classification.

### 3.5 Cross-dataset comparison of method performance

To compare the performance of each integration method across the different datasets, the distribution of ARI values was plotted using a connected box plot (Figure 6A). The boxplots summarize the distribution of ARI values for each method. The connecting lines link the ARI values obtained by each method for a given dataset, allowing visualization of the performance trends across methods within the same dataset. NEMO, SNF, and intNMF achieved the highest median ARI values (>0.350). Among all methods, only NEMO and MOFA showed ARI values over 0.500 in at least two datasets, highlighting their robustness across heterogeneous biological contexts. In contrast, moCluster exhibited the lowest median ARI value (Supplementary Table 1).

**FIGURE 6:**
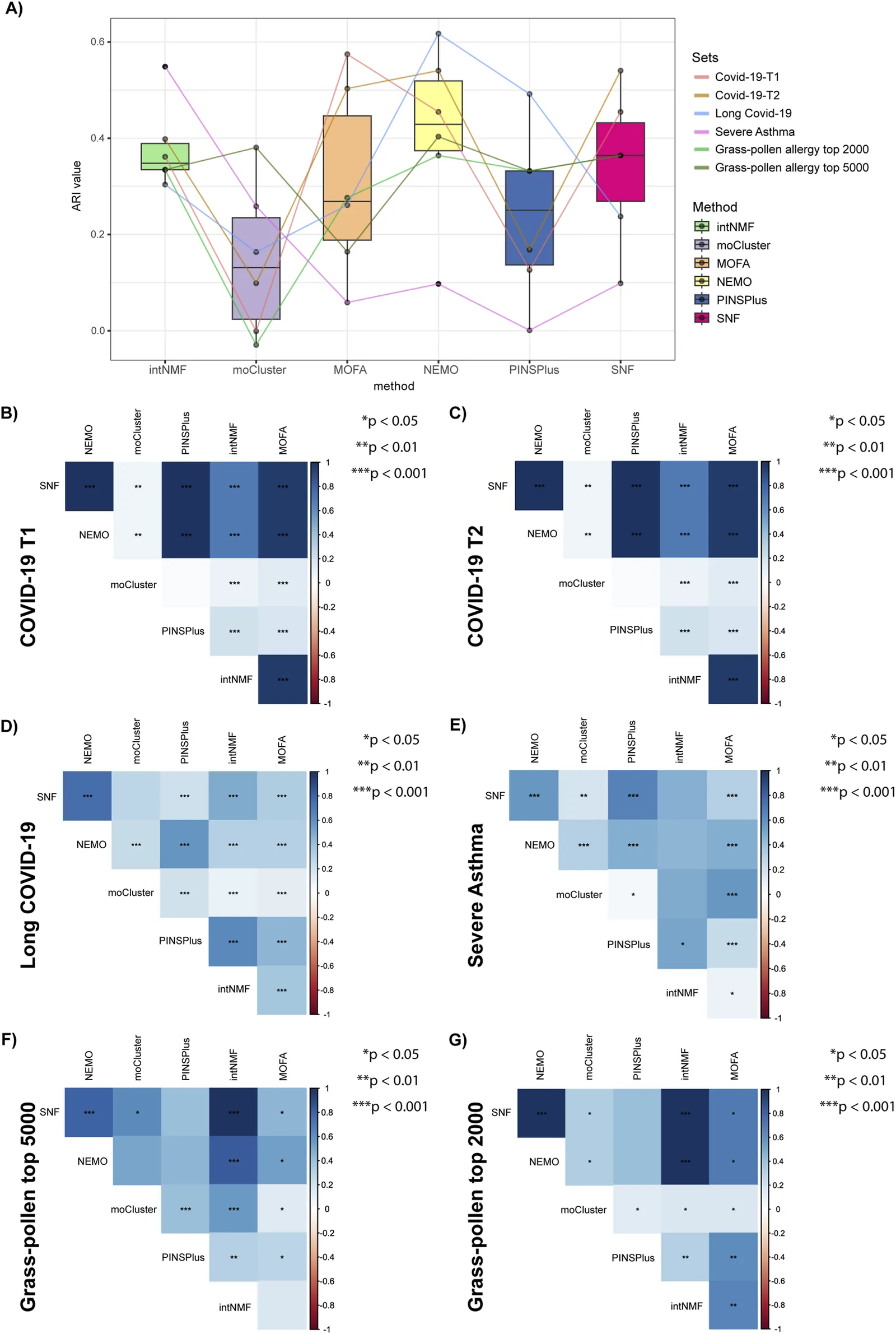
**(A)** Distribution of ARI values obtained by each integration method across all datasets, visualized using a connected box plot. Colored lines connect the ARI values corresponding to the same dataset across methods. **(B–G)** Correlation matrices based on pairwise ARI values, providing an overview of the agreement between clustering solutions generated by the different integration methods and facilitating the identification of methods showing high agreement for **(B)** COVID-19 T1 **(C)** COVID-19 T2 **(D)** Long COVID-19 **(E)** Severe Asthma **(F)** Grass-pollen top5000 and **(G)** Grass-pollen top 2000. The maximum concordance value is represented as 1 (dark blue). Corresponding p-values indicate the statistical significance of the pairwise correlations.

To evaluate the agreement among the clustering solutions generated by the different integration methods, pairwise ARI values were calculated and visualized as correlation matrices (Figures 6B–G). These matrices provide an overview of the similarity between the clustering results produced by each method and facilitate the identification of methods yielding highly concordant solutions. The statistical significance of the pairwise correlations is indicated by the corresponding p-values, which were calculated using the corrplot R package (Wei and Simko, 2024). For most datasets, SNF and NEMO produced highly similar clustering results, whereas the performance of other methods varied across datasets. In the COVID-19 datasets, PINSPlus and MOFA also showed strong concordance with SNF and NEMO (Figures 6B,C). Additionally, in the Grass-pollen allergy datasets, intNMF exhibited the highest concordance with NEMO and SNF (Figures 6F,G). In contrast, lower levels of agreement among methods were observed for the Long COVID-19 and Severe Asthma datasets (Figures 6D,E).

Based on the findings displayed above, we provide Table 2 summarizing the performance of the multi-omics integration methods in representative datasets on inflammatory respiratory diseases. These methods were classified into four performance levels (poor, moderate, good and excellent), each method’s performance dependency on dataset scale was also interpreted, and total ARI values were calculated to compare the overall performance of the methods.

**TABLE 2:** Framework for the evaluation of the integration tools.

Method	Overall precision	Best dataset size	Total ARI
SNF	Good	Scale-invariant	2.059
NEMO	Good	Scale-invariant	2.476
moCluster	Moderate	Small-scale	0.873
PINSPlus	Moderate-good	Scale-invariant	1.451
intNMF	Good	Scale-invariant	2.283
MOFA	Moderate-good	Scale-invariant	1.873

ARI, adjusted rand index; intNMF, Integrative non-negative matrix factorization; MOFA, Multi-Omics Factor analysis; NEMO, Neighbourhood-based multi-omics clustering; PINSPlus, Perturbation clustering for data INtegration and disease Subtyping; SNF, similarity network fusion.

Among the evaluated tools, NEMO has the highest total ARI (2.476), suggesting it is the optimal choice for clustering and enabling the identification of patterns among clinical groups. Moreover, intNMF ranked second (total ARI = 2.283), standing out for its consistent performance with ARI values above 0.30. Furthermore, SNF also proved solid performance across most datasets (total ARI = 2.059); however, although its algorithm is similar to that employed by NEMO and showed high agreement, NEMO ultimately delivered superior clustering results in the analyzed datasets.

## 4 Discussion

This study assessed the performance of eight widely used multi-omics integration tools across four representative datasets on inflammatory respiratory diseases. These datasets included metabolomics, proteomics, and transcriptomics data and exhibited considerable heterogeneity in terms of sample size, number of clinical groups, number of variables, and the degree of clinical distinction between groups. Such variability can substantially influence clustering performance but also reflects real-world scenarios.

A key consideration when selecting a multi-omics integration strategy is whether the study objective requires an unsupervised or supervised approach (Jovel and Greiner, 2021). Unsupervised methods are particularly useful for exploratory analyses, enabling the identification of hidden patterns, patient clustering, the generation of new hypotheses, the discovery of novel phenotypes or endotypes, the detection of outliers, and the identification of features contributing to disease heterogeneity (Zhang et al., 2023; Fukushima-Nomura et al., 2025). In contrast, supervised methods are designed to identify patterns that differentiate between predefined clinical labels and may support the discovery of novel biomarkers for diagnosis, prognosis, and/or monitoring clinical needs. In addition, supervised learning could be used to develop classification models and clinical decision-making tools that facilitate daily clinical practice (Labella et al., 2026; Sáenz De Santa María et al., 2026). This study is focused on the comprehensive assessment of unsupervised strategies across diverse datasets.

Most of the evaluated tools performed well on respiratory disease datasets regardless of sample size, except for moCluster, which yielded better clustering performance for small-sample datasets. Based on the total ARI, NEMO, intNMF, and SNF yielded the highest values. This suggests their suitability for a broad implementation across diverse inflammatory respiratory datasets. The consistent performance of NEMO across datasets highlights its potential for uncovering biologically relevant disease endotypes, which may contribute to a better understanding of phenotypic heterogeneity in allergic diseases. The identification of NEMO as the leading method for clustering has been previously documented in other studies comparing clustering algorithms on breast cancer data (Han et al., 2025). However, NEMO failed to group Severe Asthma samples, which could be related to the combination of a lower number of omics variables and the small sample size.

Furthermore, MOFA enables the identification of potential biomarkers associated with the endotypes revealed through clustering, since it reports feature-level contributions that allow for biological interpretation and comparison with previously reported biomarkers, thus supporting its translational relevance (Osama et al., 2025). Feature-level contributions are highly valuable for redefining the clinical stratifications of subjects according to their endotypes (Osama et al., 2025; Pekayvaz et al., 2024).

In addition to using four different datasets, attempting to cover the broadest possible spectrum of heterogeneity within the characteristics of the datasets and inflammatory respiratory diseases, it lacks other similar diseases, such as bacterial infections or autoimmune diseases. Beyond the disease itself, other dataset characteristics should be explored further, including comparisons between datasets of the same disease that differ in specific characteristics such as sample size, variable number, or omics technique, while keeping all other characteristics constant. This may elucidate the performance and potential bias of the methods in greater detail. Another limitation of multi-omic integration analyses lies in the lack of standardization of pre-processing protocols (Zhang and Li, 2024). Because omic layers differ in data structure, dimensionality, distribution, and frequency of missing values, each demands dedicated normalization, imputation, augmentation, and batch-effect correction to ensure proper data quality and compatibility (Lee, , 2020; Baião et al., 2025).

Beyond conventional omics layers, the integration approaches evaluated in this study could potentially be applied to other high-dimensional biological datasets. For example, allergen-resolved molecular diagnostic platforms such as ALEX2/ALEX3 generate quantitative IgE reactivity profiles for hundreds of allergen extracts and molecular components (Lupinek et al., 2025). These sensitization profiles could be incorporated as an additional data layer within a multi-omics integration framework, enabling the identification of clinically relevant patient subgroups and improving our understanding of the molecular mechanisms underlying allergic diseases. Future studies should evaluate the performance of these integration methods in such datasets.

Moreover, the incorporation of clinical data is crucial for achieving meaningful data integration and interpretation. While omics datasets provide insights into molecular mechanisms, clinical variables such as age, comorbidities, disease stage, and treatment history provide the necessary context for translating these findings into clinically relevant knowledge. In addition, several of the integration methods evaluated in this study are not restricted to molecular datasets and can accommodate diverse data modalities, including structured clinical information. Variables derived from electronic health records (EHR), patient-reported outcomes, questionnaires, laboratory tests, demographic characteristics, and disease severity assessments could be incorporated as additional data layers alongside omics measurements. Integrating molecular and clinical data may improve patient stratification, facilitate the identification of clinically meaningful subgroups, and support clinical decision-making within precision medicine frameworks (Devonshire et al., 2023). Although the integration of EHR-derived data was beyond the scope of the present study, its evaluation represents an important direction for future research.

Furthermore, clinical classification can be highly subjective, often based on criteria that may vary across practitioners or institutions. In the present study, clustering performance was assessed against predefined clinical labels, which may not fully capture the underlying biological heterogeneity of complex respiratory diseases. This limitation was particularly evident in the Severe Asthma dataset, where patient classification is extremely challenging and the results suggested the existence of additional stratifications within severe eosinophilic asthma, that may correspond to distinct biological endotypes. Consistent with this observation, clustering performance generally improved when clinically extreme groups were compared across datasets. These findings suggest that patients belonging to intermediate (i.e., moderate) disease categories may exhibit mixed phenotypes that are difficult to classify using conventional clinical criteria alone (Castro et al., 2018). Consequently, multi-omics integration may provide complementary biological information that helps refine patient stratification and supports more accurate diagnosis and treatment decisions.

Considering the valuable information that omics technologies and their integration can provide in our path towards more personalized medicine, we used a framework in this work, a framework in which, through the use of different types of data and integration tools, we were able to quantitatively assess the ability of each method to group patients, as well as evaluate the performance of the classification.

In summary, NEMO consistently outperformed the other evaluated methods in stratifying patients according to their clinical phenotypes. However, the effectiveness of multi-omics integration approaches may depend on the dataset characteristics and, in complex conditions such as inflammatory respiratory diseases, on predefined clinical classifications that may not fully capture the underlying biological mechanisms. Therefore, this work highlights the importance of considering both dataset characteristics and analytical objectives when selecting the most appropriate data integration method.

## Data Availability

The original contributions presented in the study are included in the article/Supplementary Material, further inquiries can be directed to the corresponding authors.

## Supplementary material

SUPPLEMENTARY FIGURE 1 Pre-processing workflow robustness assessment. PCA plots (A) after applying three different missing data thresholds (15%, 20%, and 25%) to the metabolomics data of the COVID-19 dataset. (B) After applying three different missing data thresholds (15%, 20%, and 25%) to the proteomics data of the COVID-19 dataset. (C) PCA plots after applying three different missing data thresholds (15%, 20%, and 25%) to the metabolomics data of the Long COVID-19 dataset. (D) PCA plots after applying three different missing data thresholds (15%, 20%, and 25%) and two different below the limit of quantification (BLQ) thresholds (25% and 35%) to the proteomics data of the COVID-19 dataset. The number of variables (n.v.) remaining in each dataset after the preprocessing is included in each plot header.

SUPPLEMENTARY FIGURE 2 Results of the COVID-19 dataset at T1. SNF heatmaps for **(A)** Mild, Moderate and Severe, and **(B)** Mild and Severe clinical groups. Each row and column represents a sample, and color intensity corresponds to pairwise sample similarity derived from Euclidean distances, with darker colors (red) indicating greater similarity. For Mild, Moderate and Severe clustering: **(C)** intNMF plot, **(D)** PINSPlus plot, **(E)** moCluster plot, **(F)** MCIA plot and **(G)** sPLS plot.

SUPPLEMENTARY FIGURE 3 Results of the COVID-19 dataset at T2 for Mild, Moderate and Severe. **(A)** NEMO heatmap, and **(B)** SNF heatmap. Each row and column represents a sample, and color intensity corresponds to pairwise sample similarity derived from Euclidean distances, with darker colors (red) indicating greater similarity. **(C)** MOFA plot, **(D)** intNMF plot, **(E)** PINSPlus plot, **(F)** moCluster plot, **(G)** MCIA plot and **(H)** sPLS plot.

SUPPLEMENTARY FIGURE 4 Results of the Long COVID-19 dataset for Control, Convalescence and Acute clinical groups. **(A)** SNF heatmap. Each row and column represents a sample, and color intensity corresponds to pairwise sample similarity derived from Euclidean distances, with darker colors (red) indicating greater similarity. **(B)** moCluster plot, **(C)** MCIA plot and **(D)** sPLS plot.

SUPPLEMENTARY FIGURE 5 Results of the Severe Asthma dataset for pre-Mepolizumab and pre-Omalizumab groups. **(A)** SNF heatmap, **(B)** NEMO heatmap, **(C)** MOFA plot, **(D)** PINSPlus plot, **(E)** MCIA plot and **(F)** sPLS plot. In NEMO and SNF, each row and column represents a sample, and color intensity corresponds to pairwise sample similarity derived from Euclidean distances, with darker colors (red) indicating greater similarity.

SUPPLEMENTARY FIGURE 6 Results of the Grass-pollen allergy top 5000 dataset. SNF heatmaps for **(A)** Control, Mild, Moderate and Severe, and **(B)** Mild and Severe clinical groups. Each row and column represents a sample, and color intensity corresponds to pairwise sample similarity derived from Euclidean distances, with darker colors (red) indicating greater similarity. **(C)** intNMF plot, **(D)** MCIA plot and **(E)** sPLS plot for Control, Mild, Moderate and Severe.

SUPPLEMENTARY FIGURE 7 Results of the Grass-pollen allergy top 2000 dataset for Control, Mild, Moderate and Severe clinical groups. **(A)** SNF heatmap, **(B)** NEMO heatmap. Each row and column represents a sample, and color intensity corresponds to pairwise sample similarity derived from Euclidean distances, with darker colors (red) indicating greater similarity. **(C)** intNMF plot, **(D)** PINSPlus plot, **(E)** MOFA plot, **(F)** moCluster plot, **(G)** MCIA plot and **(H)** sPLS plot.
