# Supplementary material for: Multi-omics integration in allergy, viral infections and asthma: a comprehensive analysis of bioinformatics tools
Source: Front Bioinform. 2026 Sep 22;6:1904588. doi: 10.3389/fbinf.2026.1904588 (PMC13638525; doi:10.3389/fbinf.2026.1904588)
Supplement: Supplementary file 1 [file Table1.docx]

**Supplementary Table 1.** Summary of the performance of the integration tools.

| DATASET | | moCluster | | PINSPlus | | intNMF | | SNF | | NEMO | | MOFA | |
| --- | --- | --- | --- | --- | --- | --- | --- | --- | --- | --- | --- | --- | --- |
|  |  | ARI | p-value | ARI | p-value | ARI | p-value | ARI | p-value | ARI | p-value | ARI | p-value |
| COVID-19 | T1 | -0.001 | 0.436 | 0.126 | <0.001 | 0.362 | <0.001 | 0.455 | <0.001 | 0.455 | <0.001 | 0.574 | <0.001 |
|  | T2 | 0.099 | <0.001 | 0.168 | <0.001 | 0.398 | <0.001 | 0.540 | <0.009 | 0.540 | <0.001 | 0.503 | <0.001 |
| Long COVID-19 | | 0.164 | <0.001 | 0.492 | <0.001 | 0.304 | <0.001 | 0.237 | <0.001 | 0.617 | <0.001 | 0.261 | <0.001 |
| Severe Asthma | | 0.259 | <0.001 | 0.001 | 0.159 | 0.549 | 0.039 | 0.099 | 0.011 | 0.097 | 0.018 | 0.059 | 0.027 |
| Grass-pollen allergy | 5000 | 0.381 | <0.001 | 0.332 | <0.001 | 0.335 | 0.003 | 0.364 | <0.001 | 0.403 | <0.001 | 0.164 | 0.017 |
|  | 2000 | -0.029 | 0.528 | 0.332 | <0.001 | 0.335 | 0.003 | 0.364 | <0.001 | 0.364 | <0.001 | 0.276 | 0.002 |
| Total ARI | | 0.873 | - | 1.451 | - | 2.283 | - | 2.059 | - | 2.476 | - | 1.837 | - |

**intNMF:** Integrative non-negative matrix factorization; **MCIA**: Multiple Co-inertia Analysis; b: matrix factorization; **MOFA**: Multi-Omics Factor analysis; **NEMO**: Neighborhood-based multi-omics clustering; **PINSPlus**: Perturbation clustering for data INtegration and disease Subtyping; **SNF**: Similarity Network Fusion; **sPLS**: sparse Partial Least Squares. *Lowest AUC calculated for any of the clinical groups.
